# Technical feasibility and short-term outcomes of single-incision endoscopic breast-conserving surgery with sentinel lymph node biopsy: initial Indian experience

**DOI:** 10.1186/s12893-025-03446-y

**Published:** 2026-03-11

**Authors:** Pon Jeeva Mathan, Keshavarajan Gandhirajan, Jagadesh Chandra Bose

**Affiliations:** Department of Surgical Oncology, Dr. Rela Hospital, Chennai, India

**Keywords:** Endoscopic breast surgery, Breast-conserving surgery, Sentinel lymph node biopsy, Minimally invasive surgery, Cosmesis, India

## Abstract

**Background:**

Endoscopic breast-conserving surgery (EBCS) aims to offer improved cosmetic outcomes while maintaining oncologic safety. Although well established in East Asian programs¹⁻³, its use remains uncommon in India⁴. This pilot feasibility series describes early Indian experience with EBCS and sentinel lymph node biopsy (SLNB) performed entirely through a single concealed axillary incision.

**Methods:**

Three consecutive women with biopsy-proven, clinically node-negative early invasive carcinoma underwent single-incision EBCS with SLNB between January and March 2025. The technique used CO₂ insufflation, a glove-port system, and ultrasonic dissection. Primary outcomes included operative parameters, margin status, sentinel node retrieval, and perioperative complications. Secondary outcomes were seroma, drain duration, pain scores, recovery milestones, and cosmetic satisfaction using a 5-point Likert scale. Follow-up was recorded individually for each patient.

**Results:**

All cases were completed endoscopically without conversion. Mean operative time was 145 min (range 130–160), and blood loss averaged 60 ml. Margins were negative in all three patients. One minor seroma resolved with conservative management. No wound infections, skin necrosis, dimpling, or lymphedema occurred. At a median follow-up of nine months, all patients remained disease-free. Cosmetic satisfaction was high (average Likert 4.6/5), with preserved breast contour and no visible deformity.

**Conclusions:**

Single-incision EBCS with SLNB is feasible, safe, and cosmetically favourable in selected Indian patients offering oncologic adequacy. While early outcomes are reassuring, larger series, longer follow-up, and validated PROMs such as BREAST-Q are required to better define the technique’s role in routine oncologic practice.

## Background

Breast-conserving surgery (BCS) followed by radiotherapy provides survival outcomes equivalent to mastectomy in early-stage breast cancer [5]. Despite its oncologic reliability, conventional BCS often results in visible scars that may affect body image and long-term satisfaction. Endoscopic and single-port techniques attempt to address these concerns by relocating incisions to concealed locations typically the axilla while preserving oncologic principles showing improved cosmetic outcomes and patient satisfaction in East Asian cohorts. Large Asian series have demonstrated reproducibility, acceptable complication rates, and favourable cosmesis [1–3], [6–8].

In India, the experience with EBCS is limited, with only isolated case reports [4], [9], [13]. This pilot series presents the earliest prospective Indian experience using a single concealed axillary incision for both endoscopic BCS and SLNB. The report focuses on detailing technical feasibility, short-term outcomes, perioperative outcomes, and early follow-up.

## Methods

### Study design and ethical approval

This prospective feasibility case series enrolled three consecutive patients between January and March 2025 at Dr. Rela Hospital, Chennai. The study was approved by the Institutional Ethics Committee (IEC/REL/ONC/2025/05). Written informed consent was obtained from all participants.

### Patient selection

Inclusion criteria were:

- Female patients ≥ 18 years.
- Invasive carcinoma ≤ 3 cm.
- Clinically node-negative (cN0).
- Unifocal tumours, preferably in the outer quadrants.
- No prior breast surgery or neoadjuvant therapy.

Exclusion criteria were multifocal or centrally located tumours, prior ipsilateral radiotherapy, or contraindications to BCS.

Baseline variables recorded included age, BMI, breast size/ptosis, tumour-to-breast ratio, depth (subcutaneous vs. retromammary), distance from the nipple, and presence of DCIS or calcifications.

### Surgical technique

**Fig. 1:**
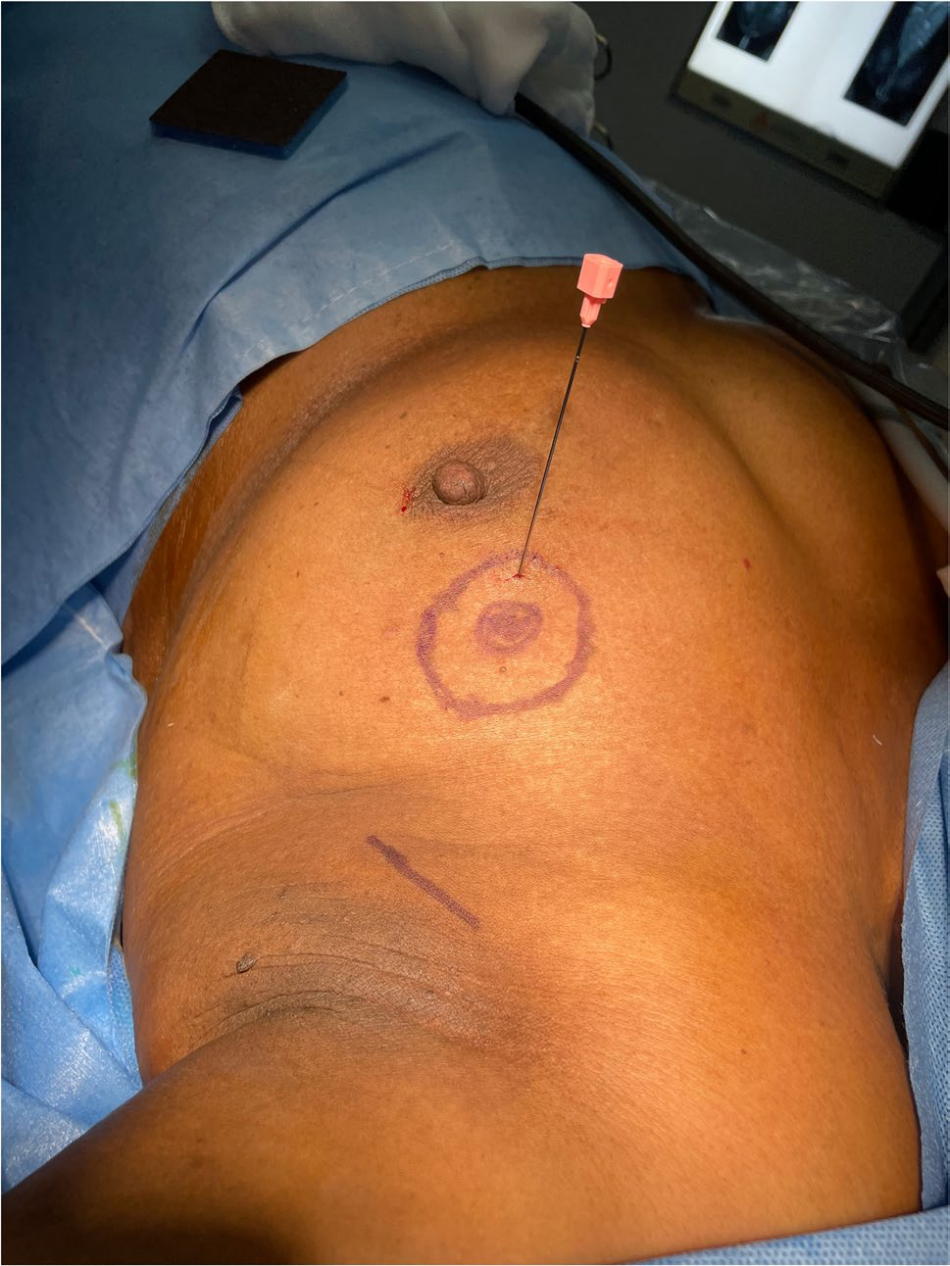
Axillary skin marking showing planned incision orientation. Localisation wire visible in situ

All procedures were conducted under general anaesthesia with the patient supine and ipsilateral arm abducted at 120°. Preoperative breast assessment is demonstrated in (Fig. 1).

**Fig. 2:**
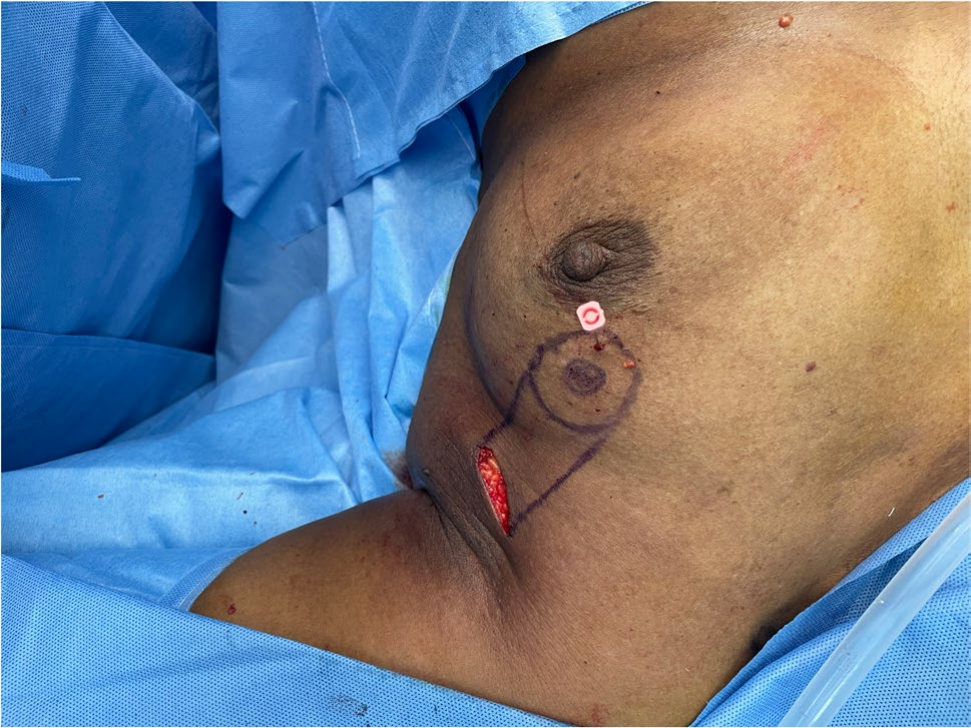
Initial axillary incision with subcutaneous dissection creating the working space for endoscopic access

**Fig. 3:**
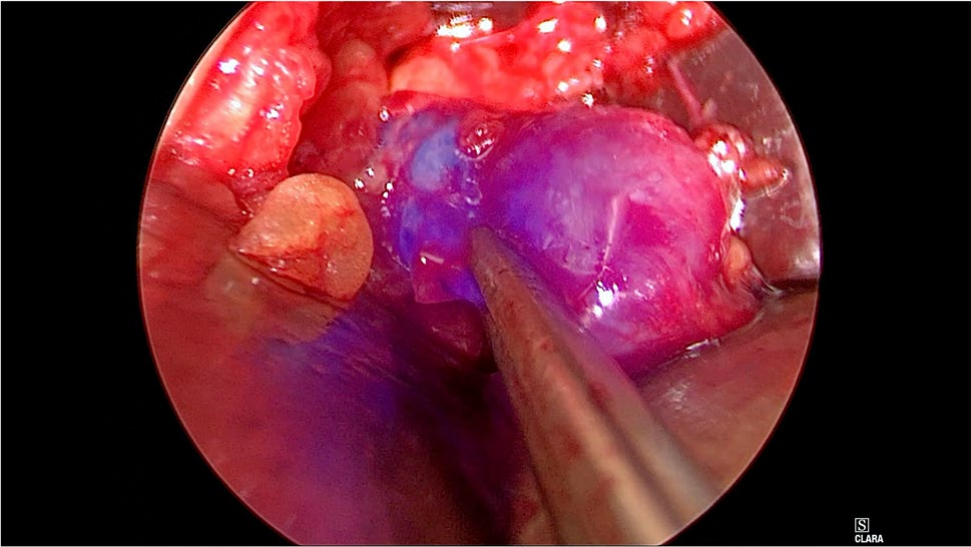
Endoscopic identification of sentinel lymph node stained with methylene blue

**Fig. 4:**
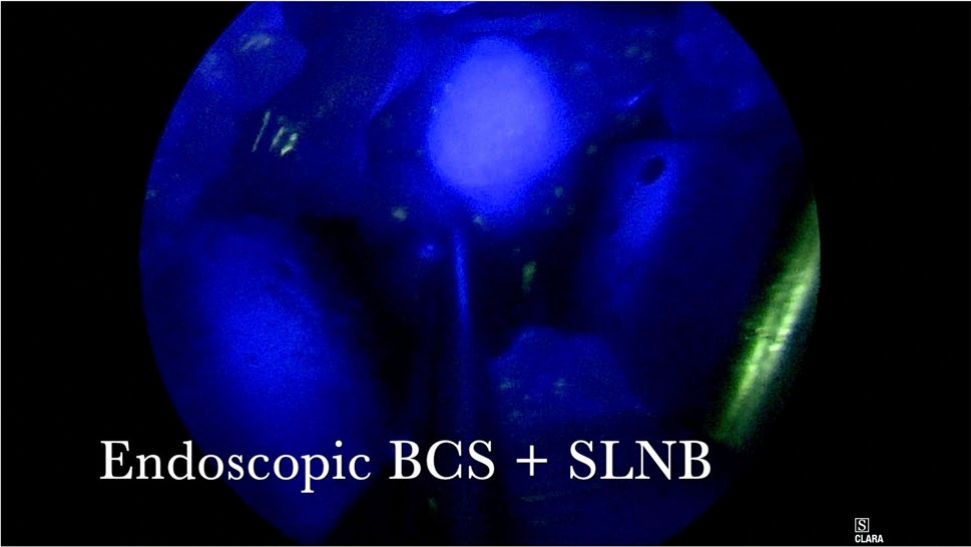
Fluorescence imaging using ICG demonstrating lymphatic drainage pathway during SLNB

A 3 to 4 cm transverse axillary incision was made can be made out in (Fig. 2). Sentinel lymph node biopsy (SLNB) employed a dual tracer protocol using indocyanine green (ICG) and methylene blue or technetium-99 m (Tc-99 m) injected peri-areolar before incision consistent with established fluorescence-guided SLNB literature [2], [8]. Endoscopic identification of the sentinel lymph node are seen in (Figs. 3 and 4). Two sentinel lymph nodes were identified and excised through the axillary incision and sent for frozen section analysis. If macro metastasis was detected that met criteria for axillary lymph node dissection (ALND), ALND was performed immediately via the same incision or extended by 1–2 cm for exposure.

**Fig. 5:**
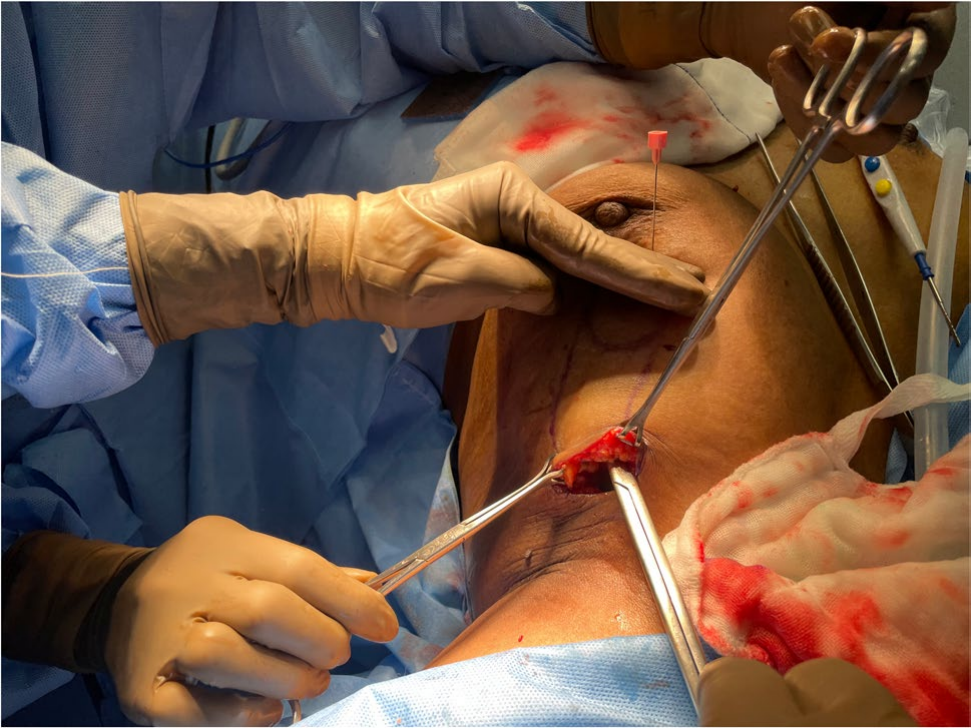
Endoscopic tunnelling technique demonstrating subcutaneous flap creation from the axillary incision toward the breast parenchyma

Hydro dissection was performed by injecting a tumescent solution containing lactated Ringer’s solution, 0.05% lidocaine, and epinephrine (1:1,000,000) into the subcutaneous plane from the nipple-areolar complex (NAC) to the periphery of the breast. Under direct vision, a subcutaneous skin flap was elevated 2–3 cm from the axillary incision to create the working space can be seen in (Fig. 5).

**Fig. 6:**
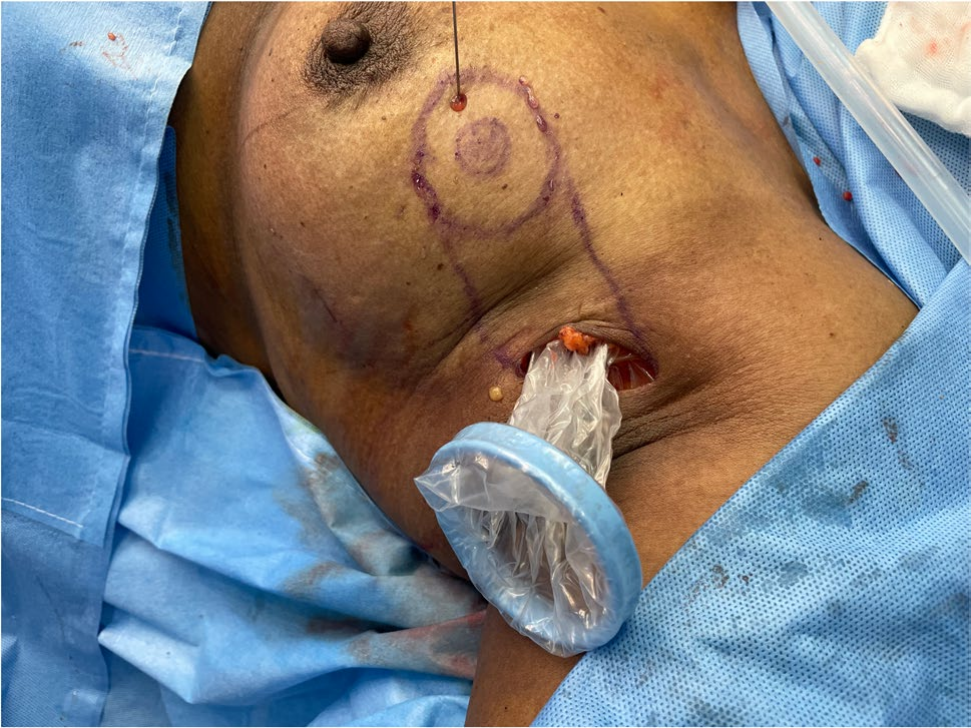
Wound protector placed within the axillary incision to maintain atraumatic access and facilitate glove-port insertion

**Fig. 7:**
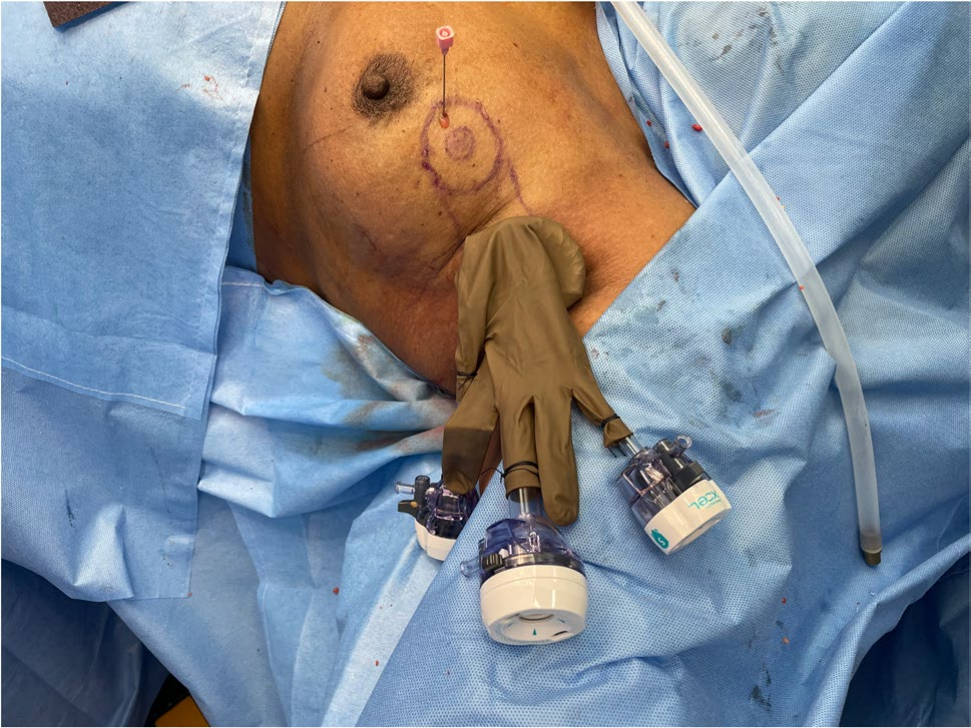
Glove‑port system assembled and inserted through the axillary incision, providing multi‑port access for EBCS

**Fig. 8:**
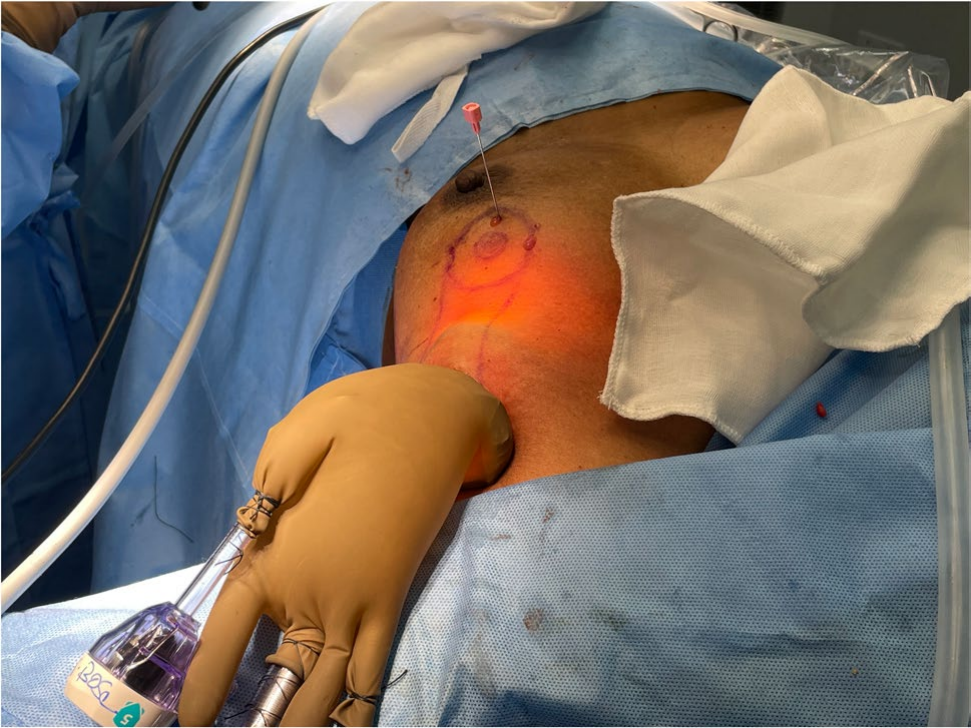
Glove-port system introduced through the axillary incision. CO₂ insufflation illuminates the subcutaneous working space, creating a uniform operative cavity for endoscopic breast dissection

**Fig. 9:**
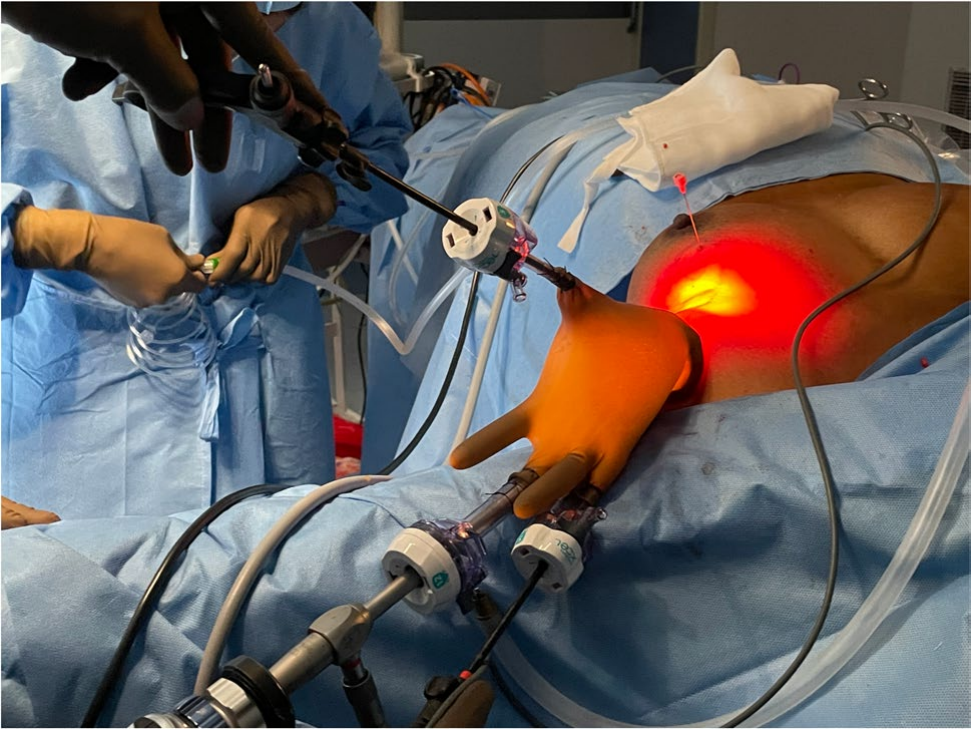
Carbon-di-oxide insufflation via glove‑port system establishing adequate endoscopic working space

**Fig. 10:**
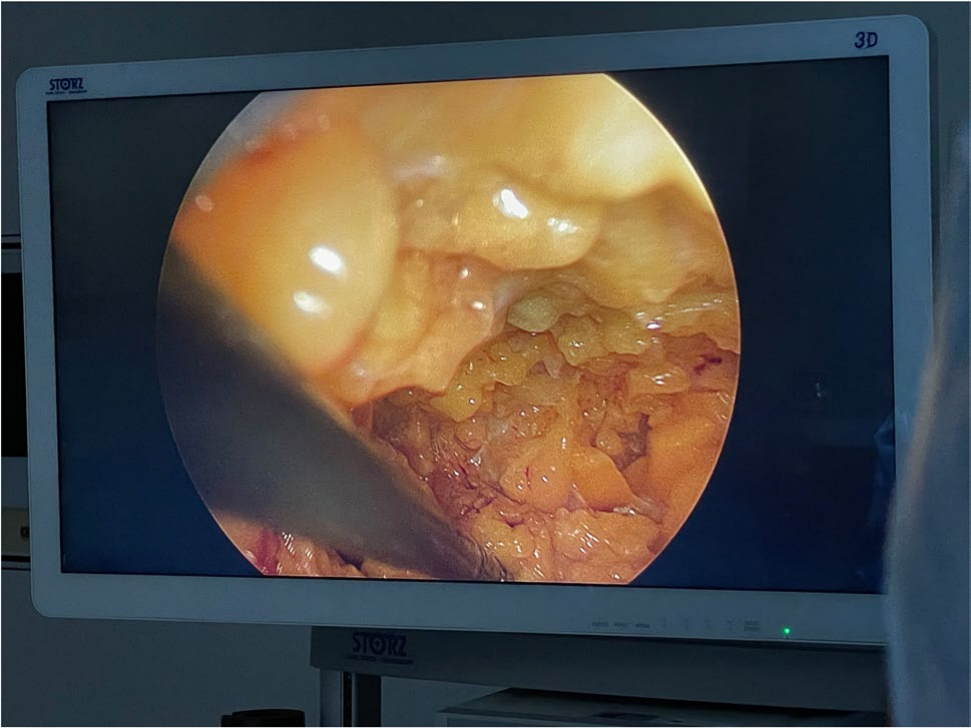
High-resolution endoscopic image showing precise dissection in the subcutaneous plane, with clear visualization of adipose tissue lobules and preserved microvascular structures

**Fig. 11:**
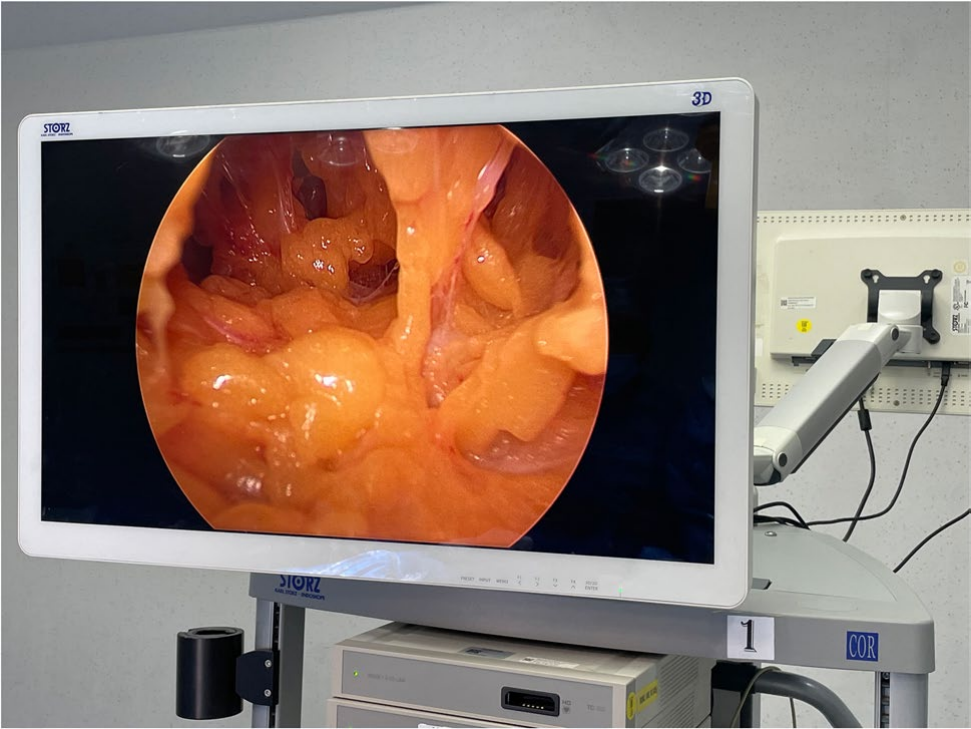
Magnified Endoscopic visualisation during tumour dissection showing preserved dissection planes

**Fig. 12:**
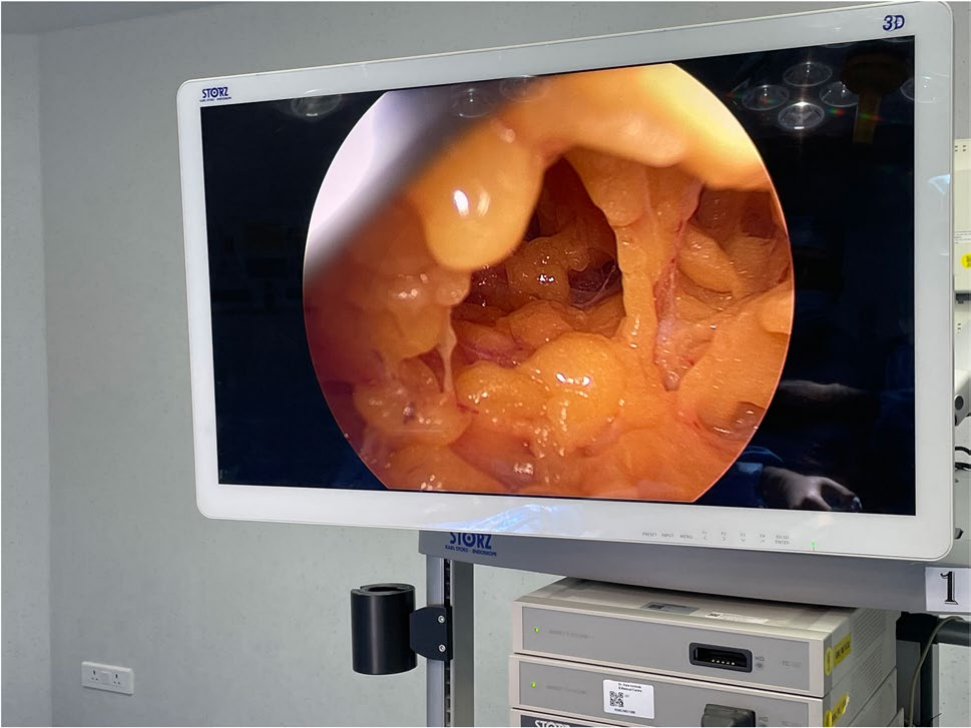
Endoscopic monitor view showing retro-glandular dissection. Fatty tissue planes are clearly visualized, allowing safe separation of the breast gland from the pectoral fascia

**Fig. 13:**
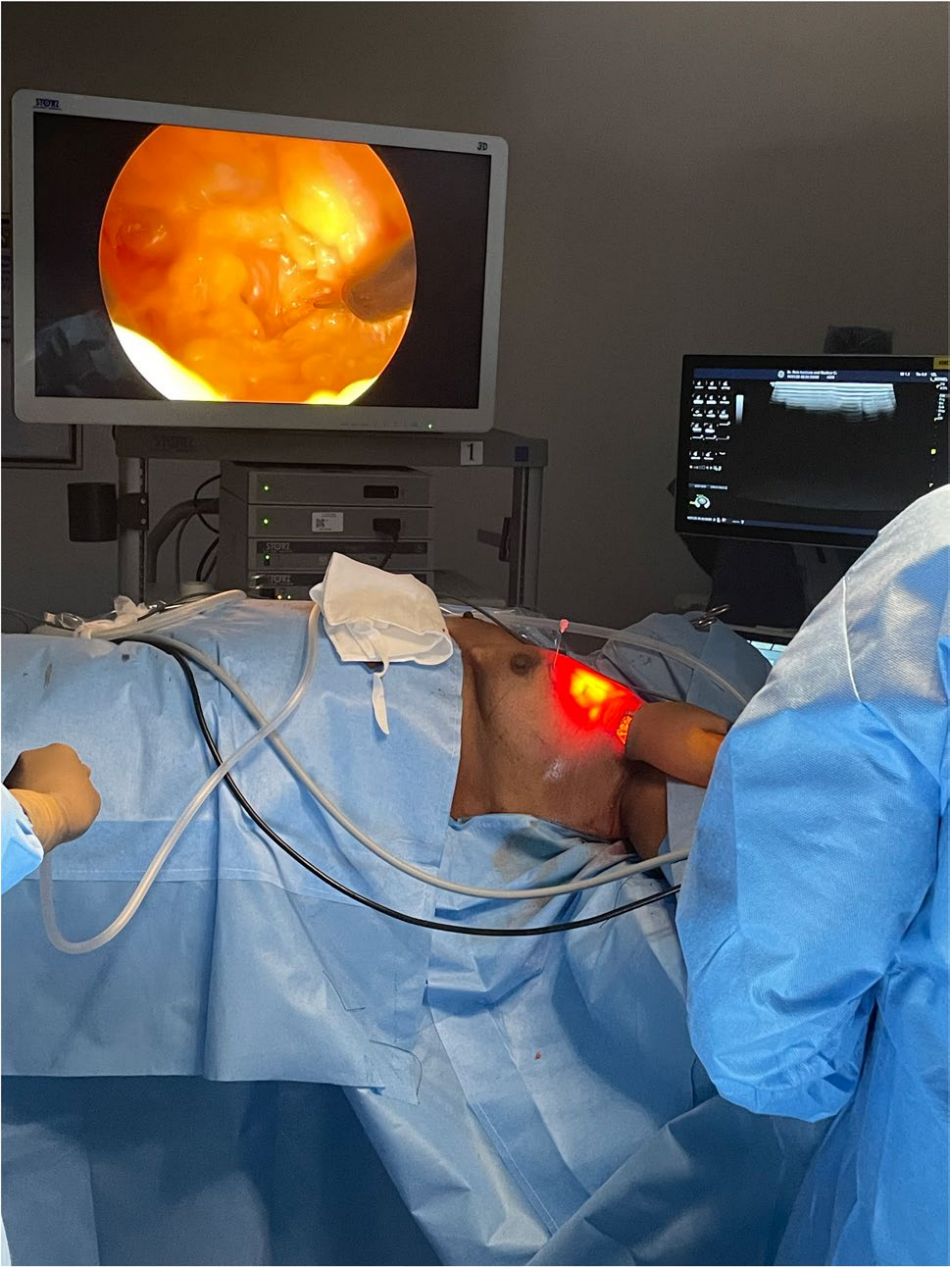
Operating room view demonstrating simultaneous endoscopic dissection and real-time screen guidance. The illuminated axillary incision highlights the insufflated cavity during tumour mobilization

A surgical glove port (Glove Port) was inserted, and carbon dioxide insufflation was maintained at 8–10 mmHg to create an adequate operative field. A 30°, 10-mm diameter 3D TIPCAM endoscope (KARL STORZ, Germany) was introduced via a 12-mm trocar. Carbon-di-oxide insufflation via glove-port system establishing adequate endoscopic working space can be seen in (Figs. 6, 7, 8 and 9). For optimal visualization, the scope was angled 30° upward with 180° reverse imaging during lateral breast dissection, then rotated downward for medial dissection. Dissection used laparoscopic diathermy curved Metzenbaum scissors with laparoscopic grasping forceps connected to suction to evacuate smoke. Intraoperative endoscopic dissection views in (Figs. 10, 11, 12 and 13).

**Fig. 14:**
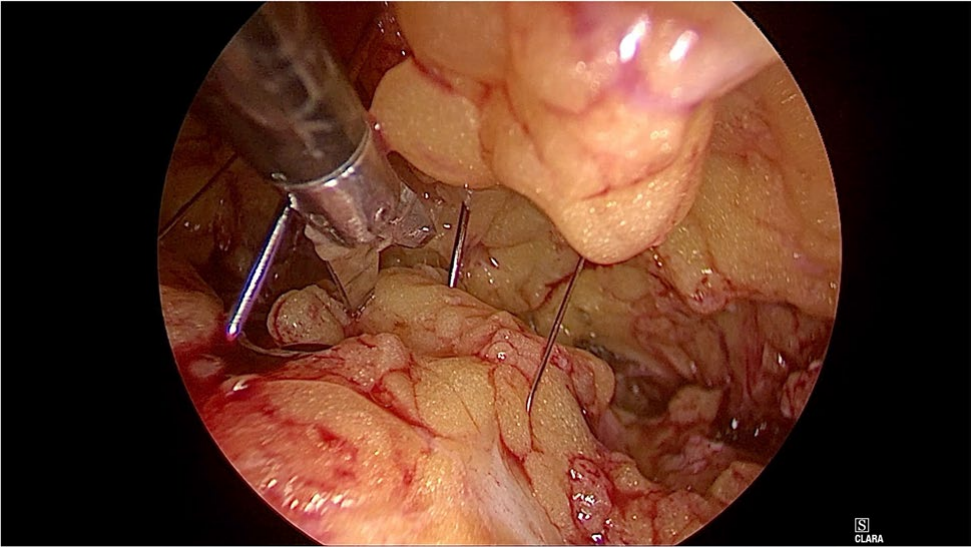
Tumor cavity with margins marked using intraoperative orientation sutures to guide pathological assessment

**Fig. 15:**
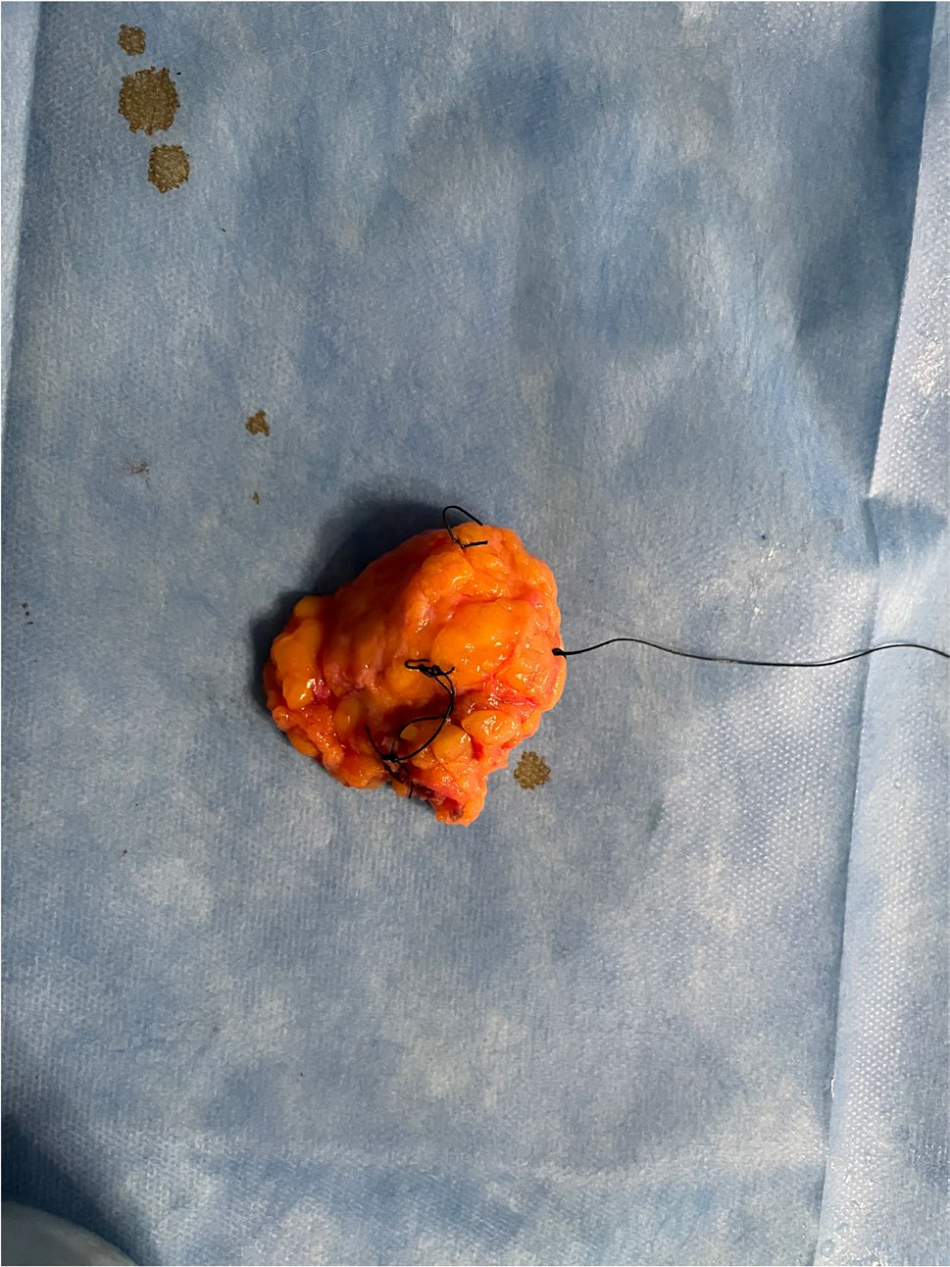
Excised tumour specimen with orientation sutures for pathological margin assessment

**Fig. 16:**
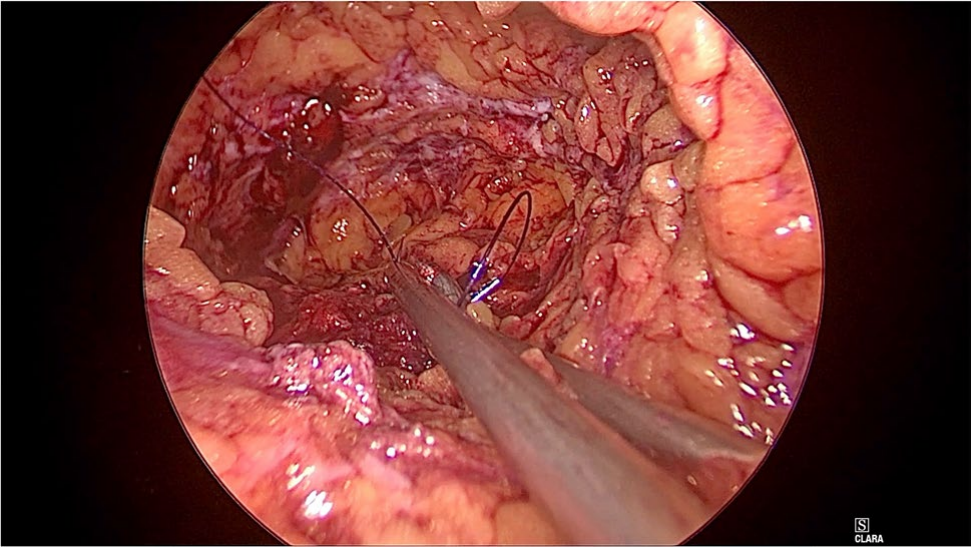
Endoscopic intracorporeal glandular suturing to restore breast contour after tumour excision

**Fig. 17:**
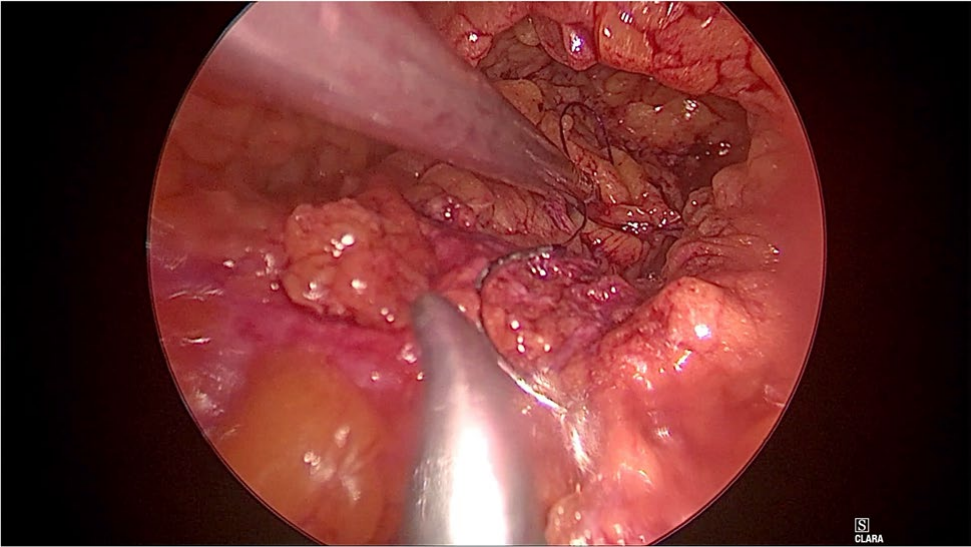
Completion of oncoplastic reconstruction showing cavity obliteration and volume restoration

The sequence began with retro glandular dissection followed by subcutaneous tunnelling guided by Metzenbaum scissors. Care was taken to avoid thermal injury to the NAC with sharp hook scissors. Tumour excision was guided by prior ultrasound needle localization and intraoperative ultrasound of the specimen can be seen in (Fig. 14). Frozen section confirmed negative margins noted in (Fig. 15). The cavity was irrigated, hemostasias secured. Endoscopic intracorporeal glandular suturing to restore breast contour after tumour excision noted in (Fig. 16). Completion of oncoplastic reconstruction showing cavity obliteration and volume restoration can be appreciated in (Fig. 17).

**Fig. 18:**
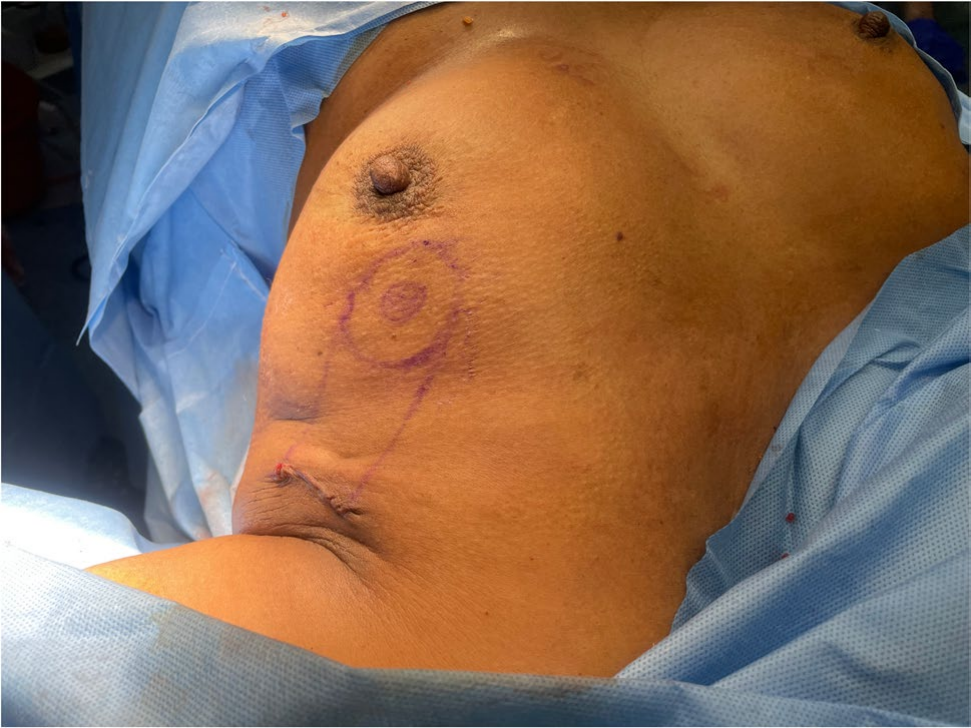
Final axillary wound closure using subcuticular suturing following EBCS and SLNB

Skin was closed with subcuticular sutures. Final axillary wound closure using subcuticular suturing following EBCS and SLNB (Fig. 18).

Specimens were retrieved using a no-touch technique within an Endo bag.

### Outcomes

Primary outcomes included:

- Operative time.
- Blood loss.
- Margin status.
- SLNB retrieval.
- Complications.

Secondary outcomes included:

- Seroma.
- Drain duration.
- Hospital stay.
- Pain scores (VAS at 24 and 48 h).
- Cosmetic outcomes (5-point Likert scale at 3 months, assessed by an independent nurse).

Validated PROMs (BREAST-Q, BCCT.core) are planned for future cohorts.

## Results

### Patient characteristics

#### Patient characteristics are summarized in table 1.

**Table 1:** Clinicopathological Characteristics

Parameter	Case 1	Case 2	Case 3
Age (years)	60	50	50
Histology	Tubular carcinoma	IDC, Grade III	IDC, Grade III
ER/PR/HER2	ER+/PR+, HER2–	ER+, PR–, HER2– (FISH–)	Triple negative
Ki-67 (%)	3–4	60–70	95
Tumour size (cm)	0.6 × 0.5	1.5 × 1.5	1.9 × 1.1 × 1.0
Sentinel nodes retrieved	2	2	2
Node status	0/2	0/2 (plus ALND)	0/2
Stage	pT1bN0	pT1cN0	pT1cN0
Adjuvant therapy	RT	CT + hormonal + RT	CT + RT

*Abbreviations*: *IDC* Invasive Ductal Carcinoma, *ER* Estrogen Receptor, *PR* Progesterone Receptor, *HER2* Human Epidermal Growth Factor Receptor 2, *FISH* Fluorescence In Situ Hybridization, *CT* Chemotherapy, *RT* Radiotherapy

**Table 2:** Comparison of EBCS vs. Conventional BCS A detailed comparison between endoscopic and conventional BCS techniques is presented in table 2

Feature	EBCS (Endoscopic)	Conventional BCS
Incision	Single concealed axillary	Periareolar/Radial/inframammary
Access	CO₂ insufflation, glove port	Open dissection
Visualization	Magnified endoscopic	Direct, limited field
Cosmesis	Hidden breast scar	Visible breast scar
Lymph node access	Same hidden incision	Separate incision or extension
Contour preservation	Good, minimal tissue distortion	Variable
Learning curve	Steep	Standard
Instrumentation	Laparoscopic/ultrasonic	Standard open instruments

### Operative & postoperative outcomes

**Fig. 19:**
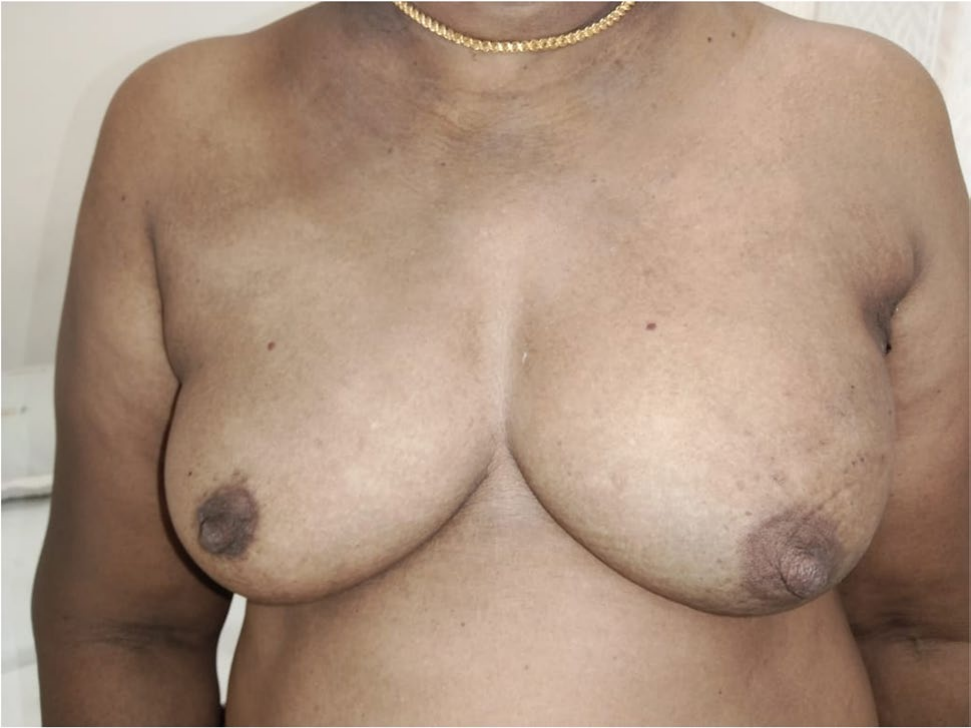
Immediate postoperative view showing concealed axillary incision and preserved breast contour

**Fig. 20:**
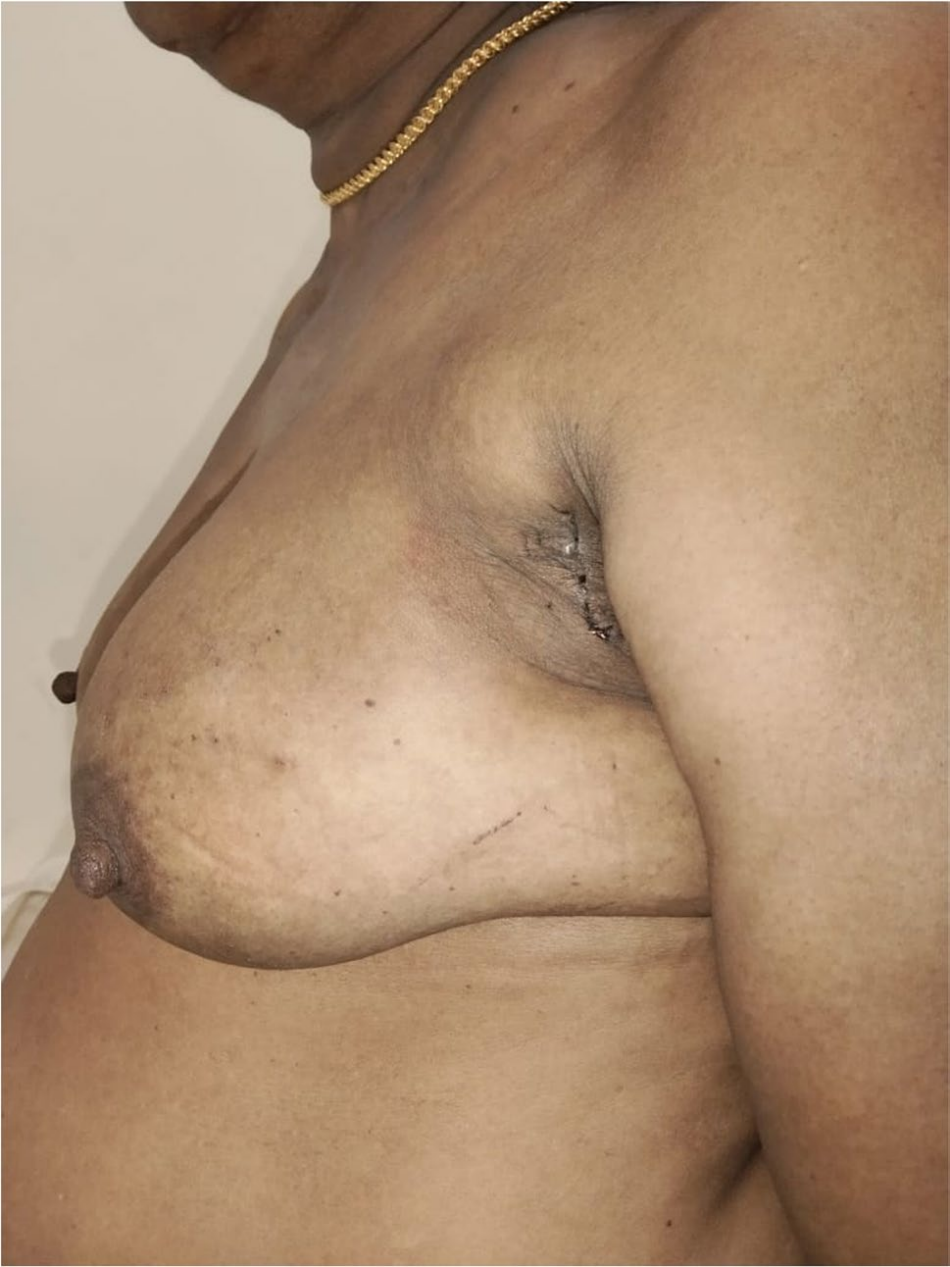
Two-week postoperative follow-up demonstrating well-healed axillary incision and maintained breast contour

**Fig. 21:**
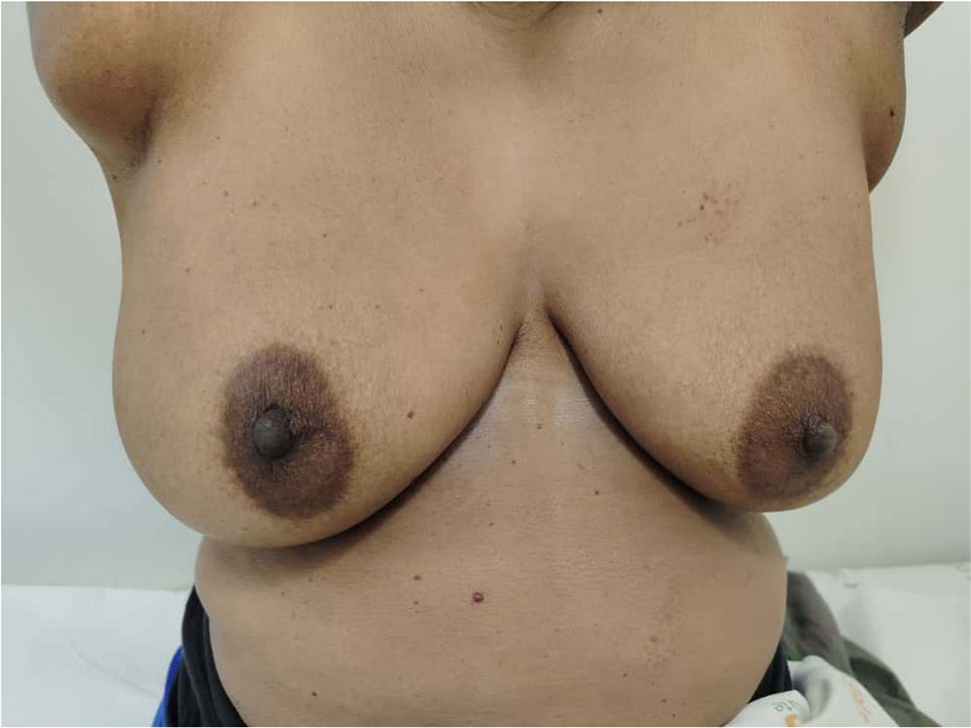
Six‑month follow‑up (frontal view) showing preserved breast symmetry, contour, and nipple–areolar position

**Fig. 22:**
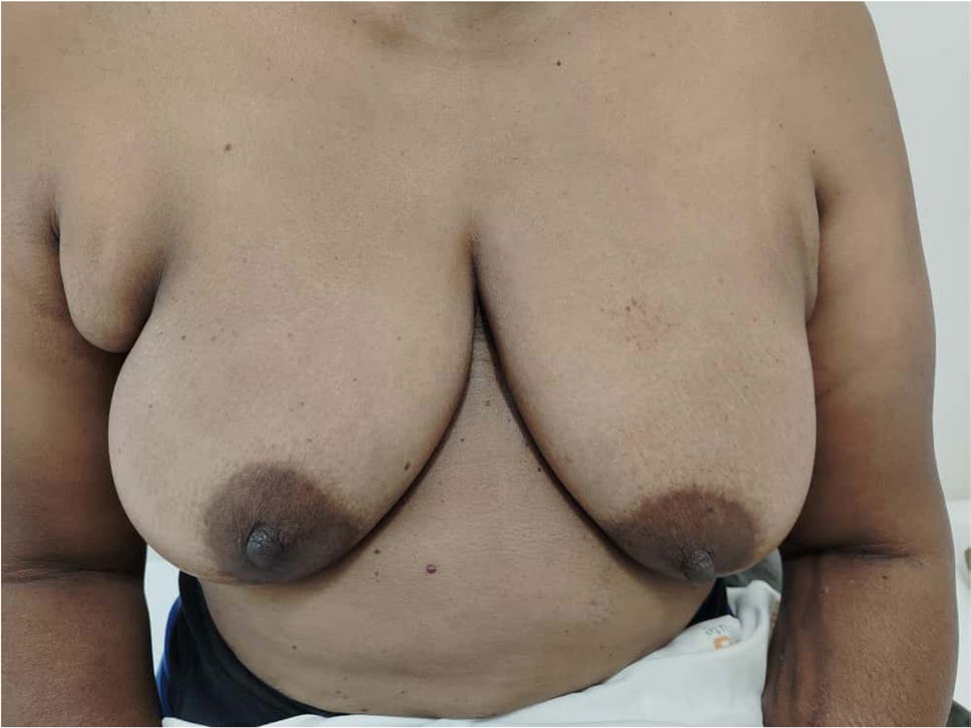
Six-month postoperative follow-up (frontal view) showing preserved breast symmetry, natural contour, and normal nipple–areolar position without visible deformity

**Fig. 23:**
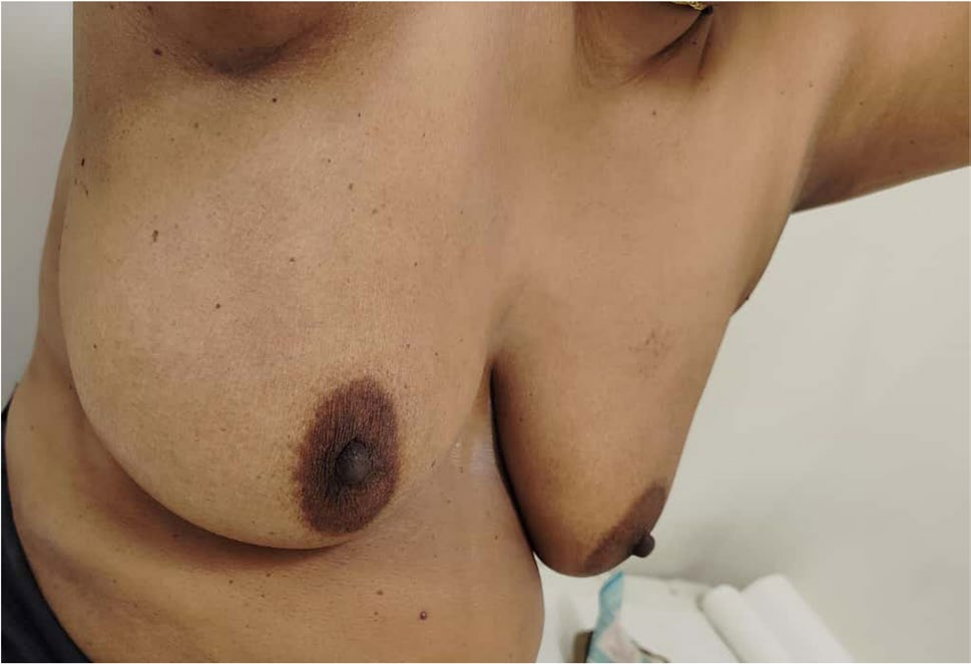
Six-month postoperative follow-up (oblique view) demonstrating maintained breast projection, smooth lower-pole contour, and absence of volume loss or contour deviation

All procedures were completed without conversion. Mean operative time was 145 minutes (range 130–160), and blood loss averaged 60 ml (range 50–70). Tumour margins were negative in all cases. One patient developed a minor seroma which was managed conservatively. No wound infections or lymphedema occurred. Median hospital stay was one day [11], [12]. Patient-reported cosmetic satisfaction was high (average Likert score 4.6/5). At a median follow-up of nine months, all patients remained disease-free. Immediate postoperative view showing concealed axillary incision and preserved breast contour in (Figs. 19 and 20). Institutional comparison with a recent cohort of ten conventional BCS cases suggested longer operative times for EBCS but similar margin clearance and complication rates. Cosmetic outcomes, including 6‑month follow-up images, are demonstrated in (Figs. 21, 22 and 23)

**Table 3:** Operative and recovery parameters operative and recovery parameters for each patient are shown in table 3

Parameter	Case 1	Case 2	Case 3
Operative time (min)	160	150	130
Blood loss (ml)	70	60	50
Margin status	Negative	Negative	Negative
Drain duration (days)	3	3	2
Seroma	No	Yes (minor)	No
Hospital stay (days)	1	1	1
Pain at 48 h (VAS)	< 3	< 3	< 3
Cosmetic score (Likert)	High	High	High
Follow-up (months)	10	8	6
Recurrence	None	None	None

## Discussion

This early Indian experience shows that single-incision EBCS with SLNB can be performed safely with reliable oncologic clearance and good cosmetic outcomes. All three procedures were completed endoscopically without conversion, and margins were negative in every case. 

Operative times were longer than conventional BCS, which is expected during the learning curve. Endoscopic magnified visualization provided precise dissection and helped maintain breast contour. The absence of visible breast scars was a key for patient satisfaction.

Our findings parallel Asian series where EBCS has been associated with high cosmetic scores and low complication rates [3], [6], [11]. Endoscopic retrieval of palpable and tracer-avid nodes through the same axillary access was straight forward, and no additional incisions were required.

Oncoplastic principles were incorporated in our series by performing intracorporeal glandular suturing to restore breast volume and maintain contour after tumour excision [10], [12]. This approach allowed us to achieve satisfactory reshaping despite the small tumour sizes. Although port-site metastasis is a theoretical concern, none were observed in our cohort, and the use of protective retrieval bags further minimizes this risk.

These findings are particularly notable within the Indian context, where published EBCS data remain scarce [4], [9], [10]. Our series contributes early evidence supporting the technique’s feasibility in Indian patients.

The outcomes of this small series are consistent with findings from major Asian centres that have established EBCS as a reproducible and aesthetically superior technique. Chia et al. reported excellent patient satisfaction and low complication rates in a large single-port cohort [6]. Nakajima and colleagues demonstrated safe margin clearance and a clear learning-curve pattern [14], while Yamashita et al. reported similarly favourable early complication and margin profiles [15]. Together, these studies support the feasibility of EBCS when performed with standardized technique and appropriate patient selection.

Robot-assisted breast surgery represents another minimally invasive approach offering superior visualization and ergonomic advantages. However, despite promising aesthetic results reported by Toesca et al. and Lai et al. [16], [17], robotic systems remain prohibitively expensive and less accessible in low-resource settings. In contrast, EBCS relies primarily on conventional laparoscopic tools already available in most Indian operating rooms, making it a more cost-effective and practical alternative.

This aligns well with India’s broader emphasis on frugal innovation, which encourages high-value, low-cost solutions to improve access to quality care [18], [19]. Our use of a glove-port system and reusable instruments contributes to significantly lower procedural costs compared with robotic or advanced single-port platforms.

Cosmetic results in our series were “subjectively excellent” at early follow-up. However, these findings should be interpreted cautiously, and future work will incorporate validated PROMs such as BREAST-Q and objective tools such as BCCT.core to provide standardized cosmetic assessment.

The SLNB performance in our patients was also acceptable, with a retrieval of two sentinel nodes per case. Evidence indicates that examining two sentinel nodes provides accurate staging while minimizing false-negative rates, with limited additional benefit beyond this number.

Although small, this initial series contributes valuable early evidence supporting the feasibility of endoscopic concealed-incision breast conservation in India, where published data remain sparse. Broader implementation will require larger multicentre studies and longer follow-up.

Individual follow-up durations reflect real-world variation, with all patients disease-free at their latest assessments.

### Limitations

- Small sample size
- Short and variable follow-up
- Technique limited initially to outer-quadrant tumours
- BREAST-Q and BCCT.core were not used in this feasibility phase

## Conclusions

Single-incision EBCS with SLNB appears feasible and safe in selected patients, offering acceptable oncologic outcomes and high cosmetic satisfaction. Larger studies with standardized PROMs and longer follow-up are needed to define broader applicability. Cosmetic outcomes at early and six-month follow-up are illustrated in (Figs. 19, 20, 21, 22 and 23). 

## Data Availability

Available from corresponding author on request.
